# The Effect of AIDS Peer Health Education on Knowledge, Attitudes, and Practices of Secondary School Students in Khartoum, Sudan

**DOI:** 10.3934/publichealth.2015.4.718

**Published:** 2015-10-22

**Authors:** Maha Hamad Mohammed Ali, Osman Babiker Osman, Mohamed AE. M. Ibrahim, Waled Amen Mohammed Ahmed

**Affiliations:** 1 Assistant Professor of Public Health, Faculty of Applied Medical Sciences, Albaha University, Al-Baha, Saudi Arabia; 2 Assistant Professor of Public Health, Faculty of Public Health, Alziem Al-Azahari University, Khartoum, Sudan; 3 Assistant Professor of Public Health, Faculty of Public and Environmental Health, University of Khartoum, Sudan. Now, Faculty of Applied Medical Sciences, Albaha University, Al-Baha, Saudi Arabia; 4 Assistant Professor of Nursing, Faculty of Applied Medical Sciences, Albaha University, Al-Baha, Saudi Arabia

**Keywords:** Peer education, HIV/AIDS, Attitude, Knowledge, Practice

## Abstract

**Background:**

Peer education seeks to enroll students in persuasive communication programs aiming at AIDS prevention. Providing information about AIDS prevention methods can lead to behavioral change and also a potential reduction in unsafe sexual behavior, particularly among young people.

**Objective:**

This study aims to assess the role of peer education interventions in improving awareness, attitudes, and practices of secondary school students and peer educators towards AIDS.

**Methods:**

This is a pre-and post-study. The study was conducted among 400 students who were randomly selected from 10 gender-balanced schools. They received the information from trained peer educators. 200 peers carried out the intervention (20 peers from each school), which was conducted in phases. The intervention required coordinating with official concerned stakeholders, preparing teaching aids, and four days of training workshops for the peer educators. The data was analyzed using the Statistical Package for Social Science program (SPSS). A paired sample t-test was obtained and utilized to interpret the changes observed in pre- and post-intervention knowledge, attitude, and practice.

**Results:**

The study showed that the intervention program improved participants' knowledge from 75.5% to 83.2%. This improvement was with specific regard to the following: the causative agent of AIDs (*p* = 0.017), which improved from 77.7% to 81.5%; the spread of HIV through mosquitos (*p* = 0.001), which showed an increase from 12.7% to 23.8%; the program focused on the concept of the HIV carrier (*p* = 0.001), and also on the AIDS risk when having multiple sex partners, (*p* = 0.001), showing an increase of 47.5% to 83.5%. Following the knowledge test, the attitudes of students significantly increased from 70% to 83% with regards to youth vulnerability to HIV (*p* = 0.001), while scored dropped from 15.7% to 8.5% concerning the topic of HIV voluntary testing (*p* = 0.001). The practices of students changed from 70% to 83% when prompted about shaking the hands of an HIV infected person and also from 84.8% to 87.7% about sharing food with an HIV infected person (*p* > 0.05).

**Conclusion:**

The study concluded that school peer education is an effective approach to inform students of unsafe sexual behavior with regards to HIV/AIDS. It is clear that peer education enables significant improvements to be made with regards to the knowledge, attitudes, and practices of the students.

## 1. Introduction

Peer education is a popular concept that implies specific approaches, communication channels, methodologies, philosophies, and strategies. The English term “peer” refers to one that is at an equal standing with others; in other words, one who belongs in an equal social group, especially regarding groups of age, gender, or status. The term “education” refers to the development, training, or persuasive knowledge resulting from an educational process [1].

HIV/AIDS peer education programs generally rely on behavioral theories. In the study, the peer education program is based partially on the Theory of Diffusion of Innovations, which assumes that an innovation could be new information, an attitude, or a practice that is thought of as new by the community [2]. Globally, peer education has been widely used as a strategy to address the HIV/AIDS pandemic as an approach in communicating behavioral change [3]. It has been widely used to meet a variety of educational objectives, and has been advocated as a potentially cheap and effective method of providing health education to pupils [4]. Peer education has been widely utilized as a prevention strategy that is accepted and valued by both program audiences and stakeholders [5]. Sexual health education for children and young adults has been the most controversial and emotional issue facing national HIV program planners and educators today [6].

Peer Education has a long history: it began being applied in health education, particularly for HIV prevention, during the 1980s. In recent years, it has been relatively popular in health education, perhaps because of the positive component of the method, which is the interaction it brings between peers [7].

Youth peer education helps to inform and train motivated young people about reproductive health and HIV issues [8]. Peer education seeks to enroll peers in persuasive communication programs that focus on HIV prevention, information, and strategies in a way that can lead to behavioral change[9] Peer education is assumed to reduce unsafe sexual behavior among young people in particular, because they are the most vulnerable group to HIV infection[9].

Such programs could be combined with other approaches, such as campaigns, mass media, youth friendly services, and school–based programs in a trial to help youth develop a sound knowledge, mature attitudes, strong beliefs, and also the necessary skills required for maintaining healthy sexual behavior [1].

There are many previous studies investigating the effects of peer education on HIV prevention among school students. For the most part, the studies come from countries that have faced HIV epidemics [10],[11]. However, there are limited studies evaluating peer interventions in HIV-afflicted areas (e.g., Sudan) where this issue is difficult to be discussed in schools.

Sudan's National AIDs program accepts peer education, as it is more effective than practices in adult education. Nevertheless, methodological difficulties and analytical problems indicate that peer education is not an easy area to investigate, with research being unable to provide definitive answers [12]. The question of this study is whether peer education is effective in improving knowledge, attitude, and practice with regards to HIV/AIDs. Thus, this study aimed to assess the role of peer education interventions in improving knowledge, attitude, and practices of the secondary school students towards AIDS.

## 2. Material & Methods

### 2.1. Study design

This study was conducted as a quasi-experimental design assessing the effect of peer education. Such a design was adopted in order to comply with studies sponsored by WHO/UNAIDs at universal and regional level[1].

### 2.2. Study area

The study was conducted in Khartoum State. The state hosted 169 female secondary schools and 151 male secondary schools with a capacity of 93,954 students (State Ministry of Education, 2010). Secondary school students represent the study population irrespective of age, race, and gender discrimination. The third level students were excluded from participation as peer educators.

### 2.3. Study population

This represents the total target (male and female students) in the study area, which is approximately 44,633 male students and 49,321 female students. The study population refers to the target schools in the study area: 169 female secondary schools and 151 male secondary schools.

### 2.4. Sampling and sample size

Multi–stage random sampling methods were adopted for the initial selection of a locality from among all the localities in the state. The seven localities were given numbers. Then, a simple, random sample (lottery method) was repeated six times, with the remainder representing the chosen locality (Karary locality).

The grand sample size was driven from the total targets (male + female) using the following formula:

n = n/1+n (e^2^)

n = total calculated sample size (400: 210 females and 190 males)

**Table 1.** Female selected schools

Name of school	Total students	Sample size
Elthora elgedida	667	64
Karary elegiga	377	36
Eldawha	225	21
Elsurorab	147	14
Elbulok	756	73
Total	2,172	210

**Table 2.** Male selected schools

Name of school	Total students	Sample size
Karary elnamothagia	400	52
The six hara	545	71
Elmushier elzipare	311	40
Gooz nafesa	060	10
Karary elegiga	133	17
Total	1,449	190

The study was conducted among 400 students who were randomly selected from 10 gender-balanced schools. They received the information from the trained peer educators. 200 peers (20 peers in each school) carried out the intervention.

Ethical clearance was obtained from the ethical committee of the Alzaiem Alazahri University, Sudan. The participants were given the right to withdraw at any stage of the study.

### 2.5. Data collection technique and instruments

The data was collected using a pre- and post-validated questionnaire, which was comprised of three parts: knowledge, attitude, and practices. The researchers and trained peers collected data from respondents at the beginning of the study, and then again after three months. The same questionnaire was used to collect data from both the secondary school students and the 20 selected peers. The pre- and post-intervention questionnaire survey was formulated and developed following the variables stated in Nigeria Peer education manual 2004[13].

### 2.6. The interventions

#### 2.6.1. Phase one

In this phase, the author contacted the official parties and stakeholders concerned, including the general director of the Ministry of Education, SNAP, and their representatives in Karary locality for the purposes of orientation, organization, coordination of efforts, legal issues, and access to educational materials.

#### 2.6.2. Phase two

The base line HIV/AIDS knowledge, attitude, and practices (KAPs) survey was conducted on over 400 students before the peer education intervention.

#### 2.6.3. Phase three: The training program of peer educators

The peers were selected according to specified criteria, which included the following: good communication and presentation skills, having a high interest, being a quick learner, and being trustworthy and respectful to colleagues. The 200 peers were then subjected to a comprehensive training involving an eight-hour workshop session held for each group of 20 peers in their own school. The aim of the training was to strengthen participants' knowledge, attitudes, and skills in peer education in order to be able to share the newly acquired knowledge and skills with their peers at school.

As adopted by the facilitators, the participatory and skilled–based teaching methods involved Sudan's National AIDs program (SNAP), trained teachers in schools, and other facilitators from the health education and public health department from the Federal Ministry of Health. The methods employed flip charts, colored and informative posters, leaflets, booklets, and a video presentation to facilitate the participatory learning techniques. This approach aimed to yield a positive outcome in the development of problem solving skills and self-confidence.

The contents include background information about STDs/HIV/AIDS as a causative agent, the routes of transmission, prevention measures, and the global and national situation concerning young people. As young people are at risk of HIV infection due to unsafe sexual practices, peer educators can positively share the concepts, principles, and responsibilities that young people should have in order to inform a wider population. As a major indicator, impact evaluation was conducted to assess the successes and failures of peer intervention in improving the knowledge, attitudes, and practices of the targeted population in the study. This evaluation was made before the students became involved in the peer interventions in the school.

#### 2.6.4. Phase four: monitoring and evaluation

The study adopted impact evaluation as a major indicator in measuring the success of peer interventions held by the trained peer students selected. In addition, a pre-test/post-test was employed three months preceding the evaluation in order to observe the changes in knowledge, attitudes and practices of the students who received or shared information with their trained peers.

### 2.7. Data analysis

The data was analyzed using SPSS (Statistical Package for Social Science). Frequency tables were formulated, converted, and finally presented as figures that showed correlations between the pre- and post-knowledge, attitude, and practices (KAPs) of students using a paired sample t-test.

## 3. Results

The study showed that the intervention program improved participants' knowledge from 75.5% to 83.2% regarding the causative agent of AIDs (*p* = 0.017); from 77.7% to 81.5% regarding the spread of HIV through mosquitoes (*p* = 0.001); from 12.7% to 23.8% on the concept of HIV carrier (*p* = 0.001), and from 47.5% to 83.5% about the increased risk of having multiple sex partners (*p* = 0.001), Table 3. The attitude of students significantly increased from 70% to 83% towards youth vulnerability to HIV (*p* = 0.001), while attitudes dropped from 15.7% to 8.5% regarding HIV voluntary testing (*p* = 0.001), Table 4. The practice of students has changed from 70% to 83% about shaking hands with an HIV infected person and from 84.8% to 87.7% about sharing food with an HIV infected person (*p* > 0.05), Table 5.

**Table 3.** Students' knowledge about issues related to HIV/AIDS before and after peer intervention

Knowledge	Correct answers before intervention (%)	Correct answers after intervention (%)	p-value
Causative agent of AIDs	75.5%	83.2%	0.017*
Spread of HIV through Mosquitoes	77.7%	81.5%	0.001*
Concept of HIV carrier	12.7%	23.8%	0.001*
Multiple sex partners increase the risk	47.5%	83.5%	0.001*

(*) significant

**Table 4.** Students' attitudes about issues related to HIV/AIDS before and after peer intervention

Attitude	Attitude before peer intervention	Attitude after peer intervention	p-value
HIV voluntary testing	84.3%	91.5%	0.001*
Youth vulnerability to HIV	70%	83%	0.001*

(*) significant

**Table 5.** Students' practice about issues related to HIV/AIDS before and after peer intervention

Practice	% Practiced before peer intervention	% Change after peer intervention	p-value
Shaking hands of HIV infected person	84.8 %	87.3%	0.07
Sharing food with HIV infected person	84.8 %	87.7%	0.06

## 4. Discussion

The results revealed an improvement in the knowledge, attitudes, and practices of secondary school students who received/shared HIV information from the trained peers. This improvement could be attributed to the intervention, which managed to create statistically significant differences with regards to the participants' attitudes.

A specific questionnaire was used to assess students' knowledge of HIV. The targeted students were initially asked about the causative agent of AIDs, and this question was repeated at the end of the peer interventions. The improvement of knowledge about the causative agents was statistically significant (*p* = 0.017). After the peer interventions, the targeted students also reported an improvement in their knowledge about the spread of HIV through mosquitoes, the concept of the HIV carrier, and how having multiple sex partners increases the risk of HIV. Following the interventions, results obtained statistically significant differences (p-values = 0.001).

These findings were similar to the findings obtained from the meta-analysis on the effectiveness of peer education interventions for HIV prevention in developing countries, which reported that using peer education interventions was associated with an improvement in HIV knowledge; specifically, the interventions were said to have caused a reduction in the sharing of injection equipment among drug users [14]. Another study conducted in Africa reported further positive changes in the knowledge of students [8]. The findings on multiple sex partners was similar to the findings that sponsored WHO and UNAIDs to assess community–based peer education programs among youth in low income countries; after analysis, the findings showed a significant reduction in the number of partners[1]. In a similar study conducted in Yemen about a school-based HIV prevention intervention among Yemeni adolescents, in which the average student had a good score of about 62.8% in HIV/AIDS transmission and prevention. Despite this, there was a statistically significant difference after peer education [15].

The students expressed positive attitudes towards voluntary testing, and the attitude was improved from 84.3% to 91.5% after peer interventions, with a statistically significant difference of *p* = 0.001. Similar findings were reported in the peer education project conducted in Africa among secondary and university peer educators and students. The study exhibited a positive role of the projects in improving the attitude of the targeted students [8]. The students' attitudes about the vulnerability of youth to HIV infection was 70% before the intervention, and increased to 83% after the peer intervention, with a markedly significant difference (*p* = 0.001). These findings were similar to the findings from the meta-analysis on the effectiveness of peer education interventions for HIV prevention in developing countries, which reported that peer education interventions were associated with improvement in HIV behaviors [14].

However, the improvement in the knowledge and attitude of the students was statistically insignificant. The level of practice on shaking hands of an HIV infected person shifted from 84.8% to 87.3% after the interventions, without statistically significant differences (*p* = 0.07). Also, attitudes regarding the sharing of food with an HIV infected person increased from 84.8% to 87.7% without showing statistically significant difference (*p* = 0.06). These results could be due to the prolonged period required for practice changes. The significant improvement in knowledge and attitude could, in the long term, lead to improvements in the practice. These findings were similar to the findings from one study conducted on Yemeni students, which showed that 54% of students targeted by peer education reported life skill changes regarding HIV/AIDs[15]. On the other hand, these findings were contrary to the findings from a study conducted in Africa, which reported positive changes in practice of the participants[8].

The strengths of the study include the use of peer interventions as a validated instrument. The limitations of the study include the lack of a control group; therefore, a fully randomized clinical trial is advised in future studies.

## 5. Conclusion

The study findings showed that the peer education is an effective approach affecting risky HIV behavior and it inflicted significant improvement in the knowledge and attitudes of students on issues related to HIV infection, but it showed insignificant changes in students' HIV related practices.
